# A Nonresonant Hybridized Electromagnetic-Triboelectric Nanogenerator for Irregular and Ultralow Frequency Blue Energy Harvesting

**DOI:** 10.34133/2021/5963293

**Published:** 2021-02-04

**Authors:** Weibo Xie, Lingxiao Gao, Lingke Wu, Xin Chen, Fayang Wang, Daqiao Tong, Jian Zhang, Jianyu Lan, Xiaobin He, Xiaojing Mu, Ya Yang

**Affiliations:** ^1^State Key Laboratory of Mechanical Transmissions, Chongqing University, Chongqing 400044, China; ^2^Key Laboratory of Optoelectronic Technology & Systems Ministry of Education, International R&D Center of Micro-Nano Systems and New Materials Technology, Chongqing University, Chongqing 400044, China; ^3^CAS Center for Excellence in Nanoscience, Beijing Key Laboratory of Micro-Nano Energy and Sensor, Beijing Institute of Nanoenergy and Nanosystems, Chinese Academy of Sciences, Beijing 100083, China; ^4^College of Aerospace Engineering, Chongqing University, Chongqing 400044, China; ^5^Shanghai Academy of Spaceflight Technology, Shanghai Institute of Space Power Source, Shanghai 200245, China; ^6^School of Nanoscience and Technology, University of Chinese Academy of Sciences, Beijing 100049, China; ^7^Center on Nanoenergy Research, School of Physical Science and Technology, Guangxi University, Nanning, Guangxi 530004, China

## Abstract

As a promising renewable energy source, it is a challenging task to obtain blue energy, which is irregular and has an ultralow frequency, due to the limitation of technology. Herein, a nonresonant hybridized electromagnetic-triboelectric nanogenerator was presented to efficiently obtain the ultralow frequency energy. The instrument adopted the flexible pendulum structure with a precise design and combined the working principle of electromagnetism and triboelectricity to realize the all-directional vibration energy acquisition successfully. The results confirmed that the triboelectric nanogenerator (TENG) had the potential to deliver the maximum power point of about 470 *μ*W while the electromagnetic nanogenerator (EMG) can provide 523 mW at most. The conversion efficiency of energy of the system reached 48.48%, which exhibited a remarkable improvement by about 2.96 times, due to the elastic buffering effect of the TENG with the double helix structure. Furthermore, its ability to collect low frequency wave energy was successfully proven by a buoy in Jialing River. This woke provides an effective candidate to harvest irregular and ultralow frequency blue energy on a large scale.

## 1. Introduction

With the depletion of petroleum energy and a series of environmental problems, searching for new sources of energy is extremely urgent in nowadays in society [1–4]. It is generally acknowledged that blue energy is able to meet the need. If used widely in commercial applications, it will bring great changes to the global energy structure, economy, and social development [5, 6]. Although the potential for ocean energy harvesting is enormous, most of the technologies for capturing this energy are still in the testing stage. In addition, most of the devices are expensive, inefficient, and so on. Therefore, new blue energy harvesting methods should become an urgent research topic [7–12].

Traditional blue energy generators are mainly based on EMGs, which require additional complex mechanical and hydraulic structures to convert wave motion into linear reciprocating or rotational motion to drive the generator to generate electricity [10, 13, 14]. This kind of devices generally has good output performance only at high frequency and in a regular environment. TENGs have many advantages, including low cost, simple structure, light weight, high power density, and random acquisition of low frequency vibration energy. [15–20]. However, most TENGs are only capable of effectively obtaining them from only one direction or within a relatively very narrow bandwidth [21–26]. For the purpose of improving the efficiency of blue energy collection, a variety of strategies is supposed to be combined to achieve the goal of working in a highly cooperative manner. A hybridized electromagnetic-triboelectric mechanism has been proven to be a convincing method to obtain blue energy [27–30].

Herein, a nonresonant hybridized electromagnetic-triboelectric nanogenerator is presented to efficiently obtain the ultralow frequency blue energy. The oscillating component of the device was supported on a fixed surface by a spring, and it can swing around this support position as a sphere, thus successfully achieving all-dimensional vibration energy obtainment. What is more, integrating the TENG with the double helix structure into the system can not only harvest energy but also effectively reduce the energy loss caused by the collision between the magnet and the outer wall, due to its elasticity as a buffer. The influences of vibration frequency on the output characteristics of the device were systematically studied based on a linear motor, and its ability to collect low frequency wave energy was proven. At the same time, the outputs under different wave heights were characterized with the action of random and irregular water waves, which proved the device's ability to collect wave energy. The results confirmed that the TENG had the potential to deliver the maximum power point at about 470 *μ*W under the loading resistance of 0.5 M*Ω* at a driving frequency of 2.2 Hz. And the electromagnetic nanogenerator (EMG) can provide 523 mW at most when the load resistance was 280 *Ω*. The energy conversion efficiency of the system reached up to 48.48%. Furthermore, we demonstrated its success on a buoy in Jialing River. Finally, the device was properly applied to powering a wireless temperature sensor, demonstrating its extensive applications toward the blue energy.

## 2. Results and Discussion

### 2.1. Model of the Hybridized Nanogenerator

**Figure 1:**
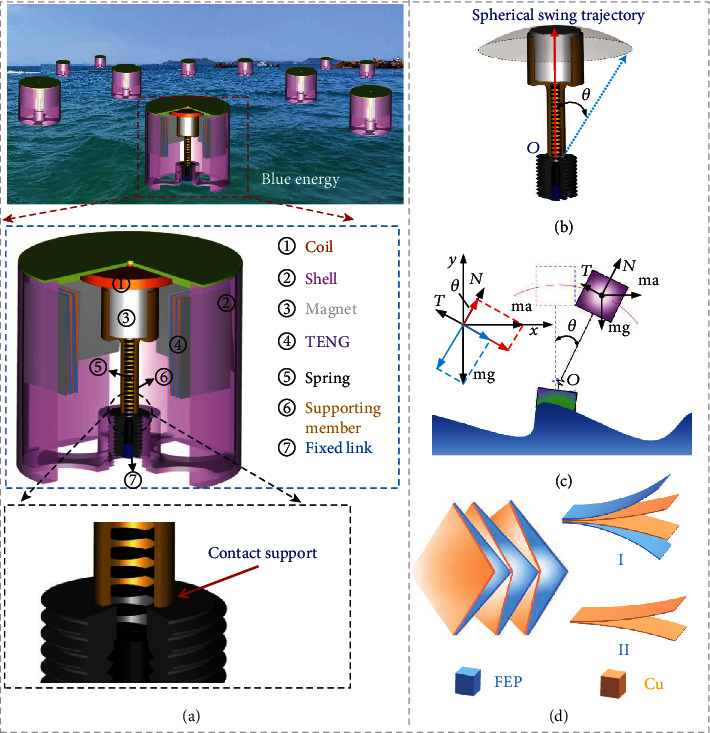
The model of the hybridized nanogenerator: (a) the schematic diagram of the device; (b) swing track diagram; (c) oscillation theory; (d) schematic diagram of the TENG.

A nonresonant hybridized electromagnetic-triboelectric nanogenerator is proposed in this work, as illustrated in Figure 1(a), which includes eight main parts: a magnet support, a cylindrical NdFeB magnet, a coil, a spring, four TENGs, a hollow cylindrical shell, an end cover, an adjusting stud, and a locking screw. The NdFeB magnet and the magnet support constituted the swing assembly, while the spring, adjusting stud, and locking screw constituted the support assembly which was installed in the inner part of the circular shell by a threaded connection. The NdFeB magnet was fixed inside the magnet support with strong glue, and the magnet support was placed on the adjusting stud after being covered on the spring, in which the adjusting stud played the role of supporting the swinging assembly and the spring was used to constrain the radial movement of the supporting position. The swing component could swing around the pivot position of the adjusting stud with a swing trajectory of a sphere, as shown in Figure 1(b). The four TENGs were uniformly spaced and pasted on the inside of the hollow cylindrical shell, and the coil was fixed on the top cover of the hollow cylindrical shell. Under the action of external excitation, with the oscillation of the oscillating component, magnetoelectric energy would be generated through the relative change of position between the magnet and coil, while triboelectric energy would be generated through the collisions between the magnet and TENGs. In order to understand the pendulum swing more intuitively, the position of the fulcrum was equivalent to the ball hinge. Movie S1 and Movie S2 are the kinematic simulation of the pendulum swing, which indicate that the swing trajectory of the pendulum is a sphere.

Taking the swing of the device to one side as an example to illustrate, the simplified model is shown in Figure 1(c). *θ* is the instantaneous angle of the oscillating component, and ma represents the resultant of the inertial force of the oscillating component and the reaction force after crashing with the shell. *N* is the supporting force, mg is the gravity force, *T* is the elastic force, and *O* is the fulcrum. Through force analysis of the swinging component, its radial force (*F*_*r*_) and tangential force (*F*_*t*_) can be given as follows:

(1) $$\begin{array}{c} F_{r}=N+\text{ma}\sin\theta -\text{mg}\cos\theta , \end{array}$$

(2) $$\begin{array}{c} F_{t}=\text{ma}\cos\theta +\text{mg}\sin\theta -T. \end{array}$$

When there is an external disturbance, such that *F*_*r*_ = 0 and *F*_*t*_ ≠ 0, the oscillating component will oscillate. For the purpose of improving the output capability of the triboelectric unit, a double helix structure triboelectric nanogenerator was employed, which we have reported in our previous study. It can not only harvest energy but also effectively reduce the energy loss caused by the collision between the magnet and the outer wall, due to its elasticity as a buffer in the system. It is constructed by two parts (part I and part II) through a novel and simple paper-folding process, as shown in Figure 1(d). Part I consisted of two pairs of copper foil (top electrode) taped together, and part II consisted of two pieces of FEP films which were attached on a copper foil (bottom electrode). In a natural state, the surfaces of part I and part II were separated because of the excellent elastic property of the double helix structure. When an external force was applied, the FEP films of part II would be in contact with the copper foil (top electrode) of part I, thus triboelectrification would be generated. The double helix structure triboelectric nanogenerator consists of five layers with the dimensions of 30 m × 30 m × 27 mm, as displayed in Figure S1.

### 2.2. Working Principles

**Figure 2:**
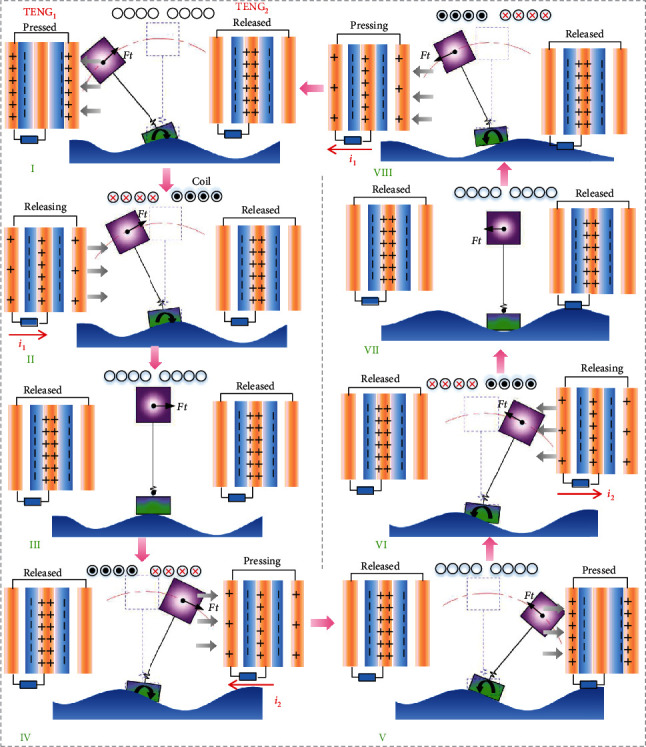
The working principle of the hybridized electromagnetic-triboelectric nanogenerator.

The example of left and right swaying was taken to demonstrate the complete operating principle of the hybridized nanogenerator, as depicted in Figure 2. Initially, the swinging part swung to the left under the action of external force and contacted with TENG_1_, assuming that no current was generated in the coil at this time (Figure 2, I). The FEP layer and the top electrode produced physical contact due to the compression of TENG_1_, so positive charges would transfer from the Fluorinated Ethylene Propylene (FEP) film to the copper film, giving rise to a mass of charges with opposite sign on each surface (Figure 2, I). When the swinging part started to swing from left to right (Figure 2, II), the top electrode was separated from the FEP film due to the elastic property of TENG_1_, resulting in a current signal from the top copper film to the bottom copper film. Meanwhile, as the magnet swung closer to the coil with the swing, the lines of magnetic induction through the coil would increase, and a clockwise current would come into being in the coil to obstruct this change. When the magnet was placed in the middle position, the top electrode was completely separated from the FEP film, and no current was generated in TENG_1_, while the magnetic flux in the coil reached its maximum value, and no induced current was generated too (Figure 2, III). When the magnet continued to swing to the right under external excitation and exerted pressure on TENG_2_, there was going to be a current signal from the bottom copper film to the top copper film in TENG_2_. Besides, a counterclockwise current was generated in the coil due to a decrease in the lines of magnetic induction through the coil (Figure 2, IV). If the magnet swung to the far right, no current was generated in TENG_2_ and the coil (Figure 2, V). When the magnet started to swing from right to left, the top electrode was separated from the FEP film under the elastic force of TENG_2_, and a current from top to bottom would be generated in TENG_2_. At the same time, the lines of magnetic induction through the coil increased, producing a clockwise induced current (Figure 2, VI). When magnet was placed in the middle position, in the same state as Figure 2, III, no current was generated in the TENG and EMG (Figure 2, VII). As the magnet continued to swing to the left and began to exert pressure on TENG_1_, there would be a same current and the counterclockwise current (Figure 2, VII). When the magnet continued to swing to the left and returned to its initial position, an energy collection cycle was completed.

### 2.3. The Output Capability of the Hybridized Nanogenerator

**Figure 3:**
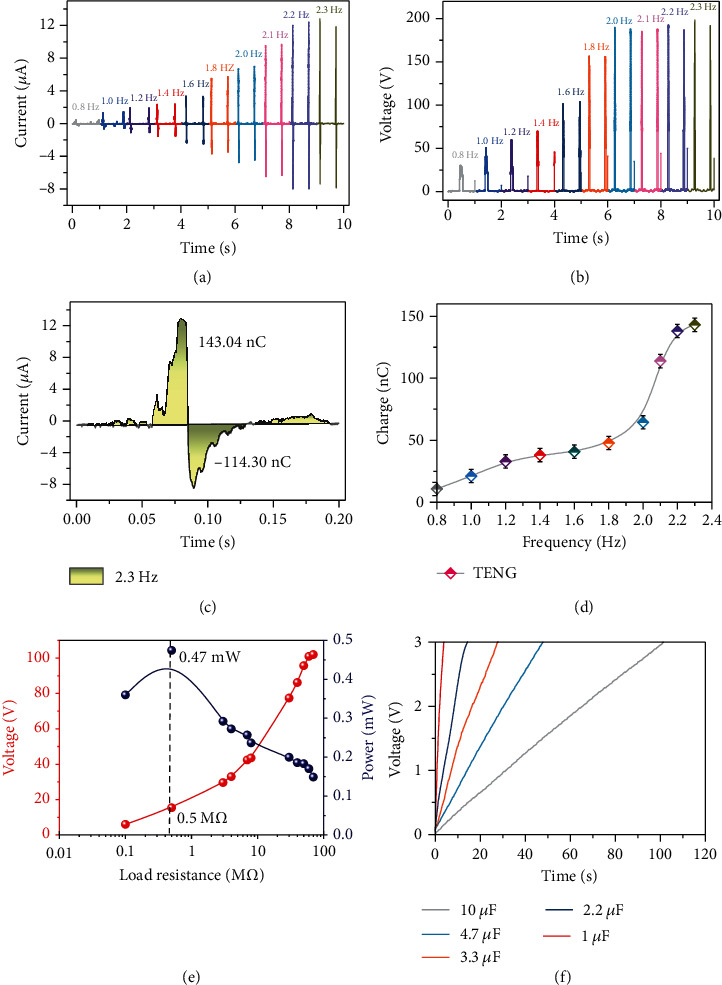
The crops of the TENG excited by a linear motor. (a) The output currents of the TENG under different vibration frequencies of external excitation. (b) The output voltages of the TENG under different vibration frequencies of external excitation. (c) The transferred charge of the TENG when the frequency of external excitation was 2.3 Hz. (d) The transferred charge of the TENG under different vibration frequencies of external excitation. (e) The peak power-resistance curve of TENG. (f) The charging behavior of the TENG.

The output characteristics were evaluated on a linear motor platform (model: DGL200-AUM4); also, oscillation frequency's influence on the output of the hybridized nanogenerator was systematically studied. Firstly, the electrometer (model: Keithley 6514) was selected to demonstrate the output capability of the TENG, as shown in Figures 3(a) and 3(b). With the frequency increasing from 0.8 Hz to 2.3 Hz, the current curves and voltage curves of the TENG show the same trend of first increasing and then stabilizing. They reached their maximum values at 2.2 Hz, 12.4 *μ*A, and 190 V. This is because vibration acceleration increases with the frequency, which will lead to the increasing of the pressure on the TENG, resulting in more triboelectric charges being on the surface. The transferred charges in one period can be obtained by integrating one period of current.  Figure 3(c) shows the current integral for TENG in one cycle at the frequency of about 2.3 Hz, and it can be seen that the transferred charges in one period are about 143.04 nC. The same method was used to calculate the transferred charges at other vibration frequencies, as shown in Figure 3(d). It indicates that the amount of transferred charge increased with the increase of the vibration frequency. In order to further verify the ability to capture the energy of the TENG, the output voltages were measured when the external load was from 1 M*Ω* to 1000 M*Ω*, and the instantaneous powers were calculated by Ohm's law. The results confirmed that the TENG has the potential to deliver a peak output power of 470 *μ*W, when the loading resistance was 0.5 M*Ω*, as presented in Figure 3(e). The charging capacity of the TENG to different capacitors was also studied, and the result shows that the voltage of an electrolytic capacitor of 10 *μ*F can be increased to 3 V within 100 seconds.

According to Faraday's law, the induced voltage in the coil is expressed as the following formula [31]:

(3) $$\begin{array}{c} V=-N\frac{d\phi }{dt}=-NS\frac{dB\left(x\right)}{dt}=-NS\frac{dB\left(x\right)}{dx}\frac{dx}{dt}=-NS\frac{dB\left(x\right)}{dx}v, \end{array}$$

where *N* is the number of windings of the coil, *S* is the area of the coil, *B*(*x*) is the magnetic field strength, and *v* is the moving speed of the magnet. When the internal resistance is *R*_coil_, the induced current in the coil is listed as

(4) $$\begin{array}{c} I=\frac{V}{R_{\text{coil}}}=-\frac{NS}{R_{\text{coil}}}\frac{dB\left(x\right)}{dx}\nu . \end{array}$$

Therefore, the output voltages and currents induced in the coil are positively correlated with the moving speed of the magnet. The magnitude and direction of the swing velocity *v* of the magnet are periodic, so is the current *I* induced in the coil. Assuming that the maximum swing angle of the magnet is *θ*, the swing angle of the magnet moving from one side to the other is 2*θ*, and the time taken is half a period, we can then calculate according to the average angular velocity of the magnet:

(5) $$\begin{array}{c} \bar{\omega }=\frac{4\theta }{T}. \end{array}$$

*T* is the period of the oscillation. Let us say the length of the pendulum is *l*, then the average velocity of the oscillation is

(6) $$\begin{array}{c} \bar{v}=\bar{\omega }l=\frac{4\theta l}{T}=4\theta lf, \end{array}$$

where *f* is the frequency of oscillation. Simultaneous Formulas (3), (4), and (6) can be obtained as follows:

(7) $$\begin{array}{c} \bar{V}=-NS\frac{dB\left(x\right)}{dx}4\theta lf, \end{array}$$

(8) $$\begin{array}{c} \bar{I}=-\frac{NS}{R_{\text{coil}}}\frac{dB\left(x\right)}{dx}4\theta lf. \end{array}$$

Therefore, the average voltage and average current induced in the coil are positively correlated with the oscillation frequency of the magnet.

**Figure 4:**
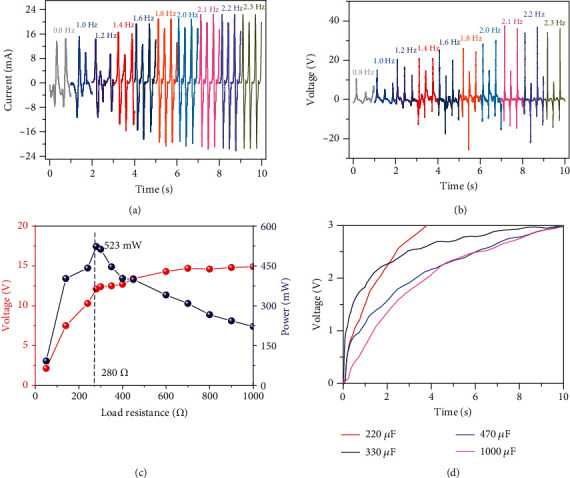
The crops of the EMG excited by a linear motor. (a) The induced currents in the coil of the EMG under different vibration frequencies of external excitation. (b) The induced voltages in the coil of the EMG under different vibration frequencies of external excitation. (c) The peak power-resistance curve of EMG. (d) The charging capability of the EMG.

The output characteristics of the EMG were evaluated on a linear motor platform, and the results are demonstrated in Figure 4. As the motion frequency of the linear motor increased from 0.8 Hz to 2.1 Hz, the maximum induced current in the coil increased from 14.50 mA to 22.45 mA, and the maximum induced voltage increased from 10.63 V to 37.42 V. The EMG represented high efficiency in the ultralow frequency range of 0.8 Hz-2.1 Hz, and the output characteristic increased with the increase of the frequency, which was consistent with the previous theoretical analysis. When the frequency was greater than 2.1 Hz, the induced current and induced voltage in the coil tend to be stable and no longer increased with the increase of the frequency. This is because the vibration frequency of the device did not rise with the increase of the external frequency due to the limitation of the structure of the device.

The relationship between the induced voltage and the impedance is shown in Figure 4(c). The relationship between peak power and impedance is obtained by calculation. It indicates that the instantaneous peak power reached its maximum of 523 mW, when the matching resistance was 280 *Ω*. 220, 330, 470, and 1000 *μ*F capacitors charged by the TEMG are shown in Figure 4(d). The voltage of an electrolytic capacitor of 1000 *μ*F can be increased to 3 V in 10 s, while the voltage of a electrolytic capacitor of 220 *μ*F can be increased to 3 V in only about 3.8 s.

### 2.4. The Wave Energy Harvesting Tests

**Figure 5:**
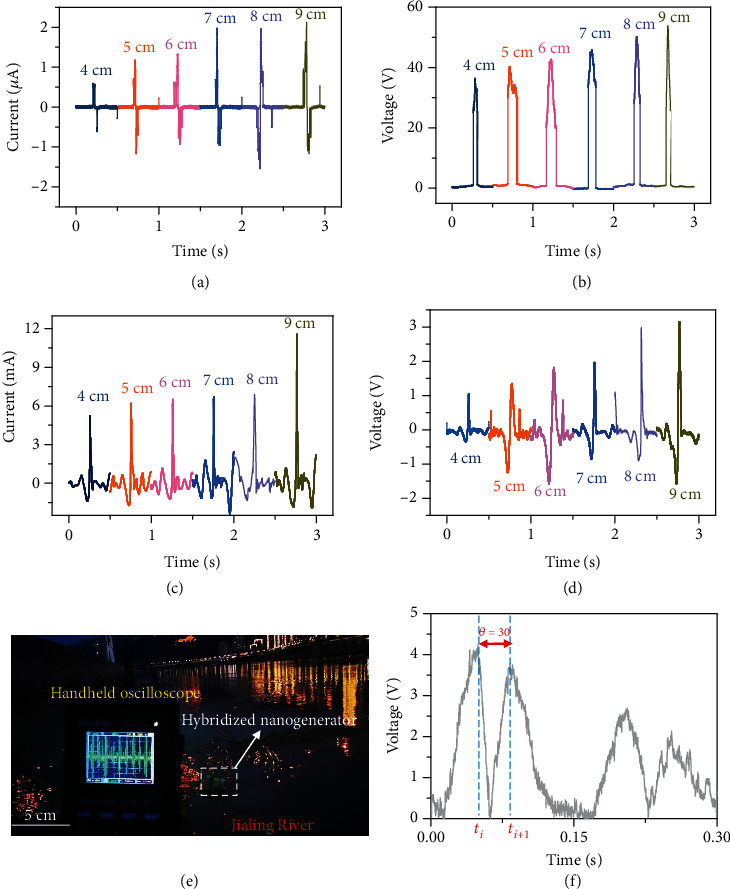
The crops of the hybridized electromagnetic-triboelectric nanogenerator in the water wave. (a) The output currents of the TENG in different wave heights. (b) The output voltages of the TENG in different wave heights. (c) The induced currents in the coil of the EMG in different wave heights. (d) The induced voltages in the coil of the EMG in different wave heights. (e) The hybridized electromagnetic-triboelectric nanogenerator on a floating buoy for water wave energy harvesting. (f) The induced voltage in the coil of the EMG under a single excitation.

Under the action of irregular and complex water waves produced by a wave pump, the outputs of a hybridized electromagnetic-triboelectric nanogenerator in different wave heights were characterized, as shown in Figure 5. Figures 5(a) and 5(b) show the output currents and output voltages of TENG at a frequency of 1.0 Hz, with wave heights ranging from 4 mm to 9 mm. The output currents of TENG improved from 0.59 *μ*A to 2.12 *μ*A and the output voltage from 36.52 V to 53.89 V with the wave height raised up from 4 cm to 9 cm, so both the output currents and output voltages of TENG showed a trend of increasing with the wave height. Similar trends were observed in the induced currents and induced voltages in the coil of the EMG, and when the wave height increased from 4 mm to 9 mm, the short-circuit current increased from 5.26 mA to 11.57 mA and the induced voltage in the coil from 1.05 V to 3.16 V, as displayed in Figures 5(c) and 5(d). In addition, a simple buoy was built to characterize the output of the hybridized electromagnetic-triboelectric nanogenerator in the Jialing River, as shown in Figure 5(e). Movie S3 explains the test of EMG in the Jialing River, and Movie S4 proves the output of TENG in the Jialing River. As shown in Figure S2, under the action of irregular and complex water waves, the peak voltages of EMG and TENG reached 93.15 mV and 60 mV, respectively.

### 2.5. The Energy Harvesting Efficiency of the Hybridized Nanogenerator

Furthermore, the captive energy efficiency of the hybridized nanogenerator was evaluated. Firstly, the energy of a single external excitation was estimated. Under the limitation of device size, the maximum swing angle of the magnet is about 30°. As can be seen from Figure 2, within the interval between two zero-current states in the coil, the device just swung 30°. Under a single excitation, the induced voltage in the coil of the EMG is shown in Figure 5(f), so the time difference between the first zero point and the second zero point can be calculated as about 0.05 seconds. Therefore, the initial angular velocity of the magnet is approximately

(9) $$\begin{array}{c} \omega =\frac{\theta }{t_{2}-t_{1}}=\frac{\pi /6}{0.05}=\frac{10\pi }{3}. \end{array}$$

The pendulum length of the device (*l*) is about 0.052 m, so the initial velocity of the magnet can be obtained through the following equation:

(10) $$\begin{array}{c} v=\omega l. \end{array}$$

The initial velocity of the magnet can be calculated to be about 0.54 m/s. Therefore, the initial energy obtained by the magnet can be obtained by the following formula:

(11) $$\begin{array}{c} E_{i}=\frac{1}{2}m\nu^{2}. \end{array}$$

The mass of the magnet (*m*) is about 0.043 kg, so the initial energy obtained by the magnet was about 6.3 × 10^−3^ J.

The electrical energy captured by the hybridized nanogenerator can be obtained through the following equation:

(12) $$\begin{array}{c} E_{o}=\int_{t1}^{t2}\frac{{U_{\text{TENG}1}}^{2}}{R_{\text{TENG}1}}dt+\int_{t1}^{t2}\frac{{U_{\text{TENG}2}}^{2}}{R_{\text{TENG}2}}dt+\int_{t1}^{t2}\frac{{U_{\text{EMG}}}^{2}}{R_{\text{EMG}}}dt. \end{array}$$

Under a single excitation, the output voltages of two TENGs under an 0.5 M*Ω* load after the integral is shown in Figure S3(a-b), and the energy they captured was 1.19 × 10^−5^ J and 1.17 × 10^−5^ J, respectively. Using the same method, under a single excitation, the output voltage of the EMG under a 280 *Ω* load after the integral is shown in Figure S3(c), and the energy it captured was 3.03 × 10^−3^ J.

Therefore, the electromechanical conversion efficiency of the hybridized nanogenerator is

(13) $$\begin{array}{c} \eta =\frac{E_{o}}{E_{i}}\times 100\%=\frac{3.054\times 10^{-3}}{6.3\times 10^{-3}}\times 100\%\approx 48.48\%. \end{array}$$

From the previous analysis, it can be seen that except for harvesting energy, the TENG also acts as a buffer layer to reduce the energy loss caused by the collision between the magnet and the outer wall. To verify this effect, the TENG was removed and the energy harvesting capability of the EMG under a single excitation was tested, as shown in Figure S3(d). In this state, the energy captured by the EMG was 1.036 × 10^−3^ J, and its electromechanical conversion efficiency was only 16.4%. It can be seen that the TENG greatly improved the energy conversion efficiency of the system.

The following is an explanation for this phenomenon based on collision theory. The power efficiency of a conventional electromagnetic generator is generally very high; however, in the system described in this paper, a lot of energy will be lost in the violent collision between the magnet and the outer wall in the narrow space during the swing process, which lowers down the most on the energy conversion efficiency of the electromagnetic generator. To settle this problem, we designed a double helix triboelectric nanogenerator of excellent resilience to save the energy loss, which also serves as another energy harvester. This sophisticated designed mechanism improved the energy conversion efficiency of the whole system. The specific collision process is shown as follows.

In this paper, the collision model of the system without the TENG can be described as Figure S4(a), in the case of ignoring the power generated by the EMG and the TENG. Because the double helix triboelectric nanogenerator can be represented by a spring model due to its excellent elastic properties, the collision model of the system with the TENG can be described as in Figure S4(b).

Collisions include elastic collisions, inelastic collisions, and completely inelastic collisions. The types of collisions can be represented by the recovery coefficient *e*:

(14) $$\begin{array}{c} e=\frac{v_{2}-v_{1}}{v_{10}-v_{20}}, \end{array}$$

where *v*_10_ is the speed of the magnet before collision, *v*_1_ is the speed of the magnet after the collision, *v*_20_ is the speed of the outer shell before collision, and *v*_2_ is the speed of the outer shell after the collision. When *e* = 1, it corresponds to the elastic collision, that is, after the collision, the deformation can recover; there is no heat, no sound, and no kinetic energy loss. When *e* = 0, it corresponds to a completely inelastic collision, that is, after the collision, the objects are combined together, or the velocities are equal, and the kinetic energy loss is the greatest. When 0 < *e* < 1, it corresponds to inelastic collision, that is, the object cannot completely recover to its initial state after collision.

In this paper, for the models in Figure S4(a) and Figure S4(b), the velocities of the outer shell were zero before and after the collision:

(15) $$\begin{array}{c} v_{2}=v_{20}=0. \end{array}$$

Therefore, Formula (15) can be simplified as

(16) $$\begin{array}{c} e=-\frac{v_{1}}{v_{10}}. \end{array}$$

The kinetic energy of the magnet before collision was

(17) $$\begin{array}{c} E_{1}=\frac{1}{2}{{mv}_{10}}^{2}. \end{array}$$

The kinetic energy of the magnet after collision was

(18) $$\begin{array}{c} E_{2}=\frac{1}{2}{{mv}_{1}}^{2}. \end{array}$$

Therefore, the energy loss after the collision was

(19) $$\begin{array}{c} \Delta E=\frac{1}{2}{{mv}_{10}}^{2}-\frac{1}{2}{{mv}_{1}}^{2}=\frac{1}{2}{{mv}_{10}}^{2}\left(1-e^{2}\right). \end{array}$$

In the model which is shown in Figure S4(a), the magnet would make a huge noise during the direct collision with the outer shell (white resin). At the same time, the elastic property of the white resin was poor. After deformation, it was not easy to recover, so it was an inelastic collision. Namely, 0 < *e* < 1 and the energy loss after collision was

(20) $$\begin{array}{c} \Delta E=\frac{1}{2}{{mv}_{10}}^{2}\left(1-e^{2}\right)\cdots \left(0<e<1\right). \end{array}$$

As for the model shown in Figure S4(b), because the spring had excellent recovery performance, it can be approximately equivalent to the elastic collision, which was *e* = 1, and the collision energy loss was zero.

Therefore, the energy loss in the collision process can be effectively reduced, and the energy conversion efficiency of the system can be increased after integrating the double helix structure triboelectric nanogenerator on the inner wall of the system.

### 2.6. The Application of the Hybridized Nanogenerator

**Figure 6:**
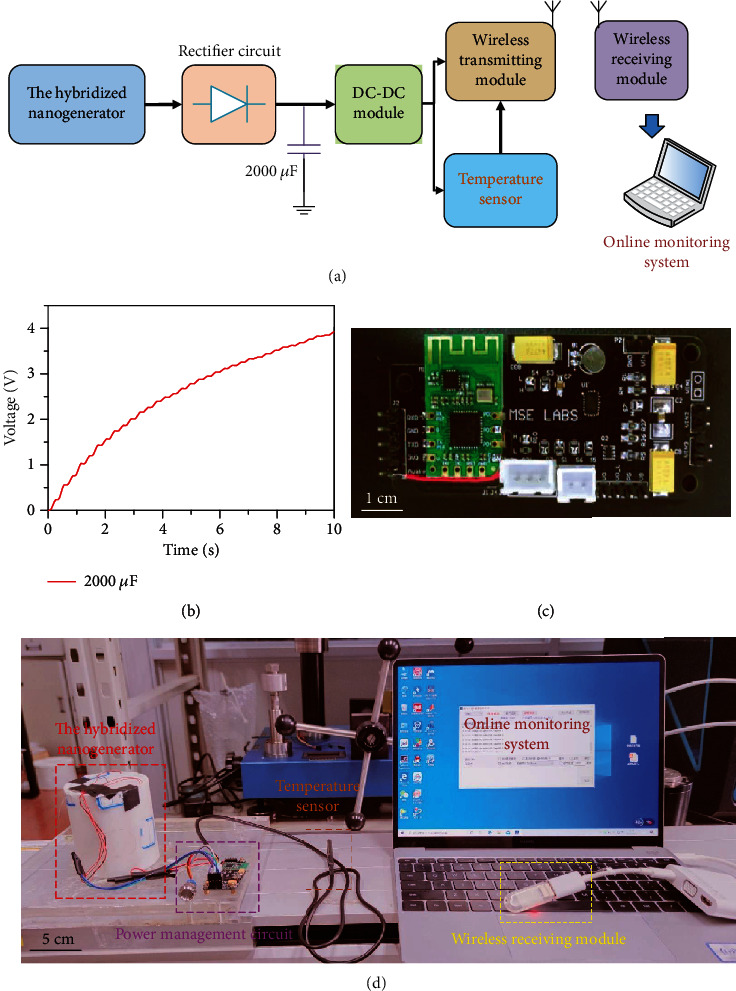
The self-powered wireless temperature sensing system. (a) The schematic diagram of the application system. (b) The charging performance of the hybridized nanogenerator for a capacitor of 2000 *μ*F. (c) Photograph of the power management circuit. (d) Photograph of the application system.

On the linear motor, lighting LED experiments had been accomplished. In Figure S5(a-b) and Movie S5-6, 100 LEDs in parallel were lighted up simultaneously by the EMG; besides, 50 LEDs in series were lighted up by the TENG. Finally, a self-powered wireless temperature sensing system was successfully integrated with a simple power management module, and the system block diagram is shown in Figure 6(a). Figure 6(b) displays the voltage of an electrolytic capacitor of 2000 *μ*F which could be raised up to 4 V in about 10 seconds by the hybridized nanogenerator. The core management component of the power management module was the LTC3106 made by the ADI company for energy acquisition and power management. The temperature sensor is a K-type thermocouple, whose measurement range is 0-200°C. The main control chip of the wireless transmission module is CC2530F256, and its longest distance of transmission is 600 m. The electronic photos of the power management are depicted in Figure 6(c). During the testing phase, we added a switching element at the front end of the wireless temperature sensor. Firstly, turning off the switch, the hybridized nanogenerator charged a rechargeable battery of 1 mAh through the input capacitor, and the charging protection voltage was 3 V. After charging for a period of time, the switch was turned off, and the energy of the hybridized nanogenerator was transferred from the input capacitor to the wireless temperature sensing module through the DC/DC module, where the excess energy was stored in the rechargeable battery. Wireless temperature data can be displayed in the serial port program via the USB to serial port. The system photo is shown in Figure 6(d). Experiments show that the hybridized nanogenerator can successfully support the wireless temperature sensing to complete data transmission with an interval of 10 seconds, as shown in Move S7.

## 3. Conclusions

Overall, a nonresonant hybridized electromagnetic-triboelectric nanogenerator was presented to efficiently harvest the ultralow frequency blue energy. Through the circumferential swing structure design, the low frequency, random, and irregular vibration energy harvesting was achieved successfully. On the basis of a linear motor platform and wave pump, this paper systematically studied the influence of oscillation frequency and wavelength. The results confirmed that the triboelectric nanogenerator (TENG) had the potential to deliver the maximum power point of about 470 *μ*W while the electromagnetic nanogenerator (EMG) can provide 523 mW at most. The energy conversion efficiency of the system was successfully improved to 48.48% due to the elastic buffering effect of the TENG with the double helix structure. Furthermore, its effectiveness for wave energy harvesting was verified on a simple float in the Jialing River. Finally, the device was proven to successfully create a self-powered wireless temperature sensing system, demonstrating its extensive applications toward blue energy.

## 4. Materials and Methods

### 4.1. Fabrication of the Hybridized Nanogenerator

The shell, magnet support, and adjusting stud were printed with white resin by 3D-printing technology. The size of hollow cylindrical shells was *Φ*86 mm × 80 mm, and the size of the magnet was *Φ*22 mm × 20 mm. The magnet was fixed inside the shell by a spring with a line diameter of 0.5 mm, an outside diameter of 5 mm, and a height of 40 mm. A 3D-printed hollow rod was placed on the outside of the spring to increase its stiffness. Then, the hollow rod was fixed at the lower end of the shell by an adjusting stud. Finally, four TENGs were attached on the inner wall of the shell, and a coil of 3000 turns with a size of *Φ*40 mm × 4 mm was attached to the lid of the shell.

### 4.2. Characterization

The outputs of the device were acquired by a programmable electrometer whose model was the Keithley model 6514. The external vibrations were excited by a DGL200-AUM4 and a wave pump. The outputs of the hybridized nanogenerator in the Jialing River were acquired by a Hantek 2C42.

## Additional Points

*Research Data*. The (xlsx) data used to support the findings of this study are included within the supplementary information (shared data).

## Supplementary Materials

Supplementary Materials Figure S1: pictures of double helix structure triboelectric nanogenerator. Figure S2: hybridized nanogenerator in Jialing River. Figure S3: hybridized nanogenerator's energy harvesting efficiency. Figure S4: the collision models of the system without TENG and with TENG. Figure S5: LEDs lighted up by hybridized nanogenerator. Movie S1: the front view of the kinematic simulation for pendulum swing. Movie S2: the vertical view of the kinematic simulation for pendulum swing. Movie S3: the outputs of EMG tested in the river. Movie S4: the outputs of TENG tested in the river. Movie S5: 100 LEDs in parallel were lighted up by the EMG. Movie S6: 50 LEDs in series were lighted up by the TENG. Movie S7: The wireless temperature sensing was powered by the hybridized nanogenerator. Shared data 1: Figure 3(a) data. The output currents of the TENG under different vibration frequencies of external excitation. Shared data 2: Figure 3(b) data. The output voltages of the TENG under different vibration frequencies of external excitation. Shared data 3: Figure 3(c) data. The transferred charge of the TENG when the frequency of external excitation was 2.3 Hz. Shared data 4: Figure 3(d) data. The transferred charge of the TENG under different vibration frequencies of external excitation. Shared data 5: Figure 3(e) data. The peak power-resistance curve of TENG. Shared data 6: Figure 3(f) data. The charging behavior of the TENG. Shared data 7: Figure 4(a) data. The induced currents in the coil of the EMG under different vibration frequencies of external excitation. Shared data 8: Figure 4(b) data. The induced voltages in the coil of the EMG under different vibration frequencies of external excitation. Shared data 9: Figure 4(c) data. The peak power-resistance curve of EMG. Shared data 10: Figure 4(d) data. The charging capability of the EMG. Shared data 11: Figure 5(a) data. The output currents of the TENG in different wave heights. Shared data 12: Figure 5(b) data. The output voltages of the TENG in different wave heights. Shared data 13: Figure 5(c) data. The induced currents in the coil of the EMG in different wave heights. Shared data 14: Figure 5(d) data. The induced voltages in the coil of the EMG in different wave heights. Shared data 15: Figure 5(f) data. The induced voltage in the coil of the EMG under a single excitation. Shared data 16: Figure 6(b) data. The charging performance of the hybridized nanogenerator for a capacitor of 2000 *μ*F.
