# Supplementary material for: A Nonresonant Hybridized Electromagnetic-Triboelectric Nanogenerator for Irregular and Ultralow Frequency Blue Energy Harvesting
Source: Research (Wash D C). 2021 Feb 4;2021:5963293. doi: 10.34133/2021/5963293 (PMC7881767; doi:10.34133/2021/5963293)
Supplement: Supplementary Materials — Figure S1: pictures of double helix structure triboelectric nanogenerator. Figure S2: hybridized nanogenerator in Jialing River. Figure S3: hybridized nanogenerator's energy harvesting efficiency. Figure S4: the collision models of the system without TENG and with TENG. Figure S5: LEDs lighted up by hybridized nanogenerator. Movie S1: the front view of the kinematic simulation for pendulum swing. Movie S2: the vertical view of the kinematic simulation for pendulum swing. Movie S3: the outputs of EMG tested in the river. Movie S4: the outputs of TENG tested in the river. Movie S5: 100 LEDs in parallel were lighted up by the EMG. Movie S6: 50 LEDs in series were lighted up by the TENG. Movie S7: The wireless temperature sensing was powered by the hybridized nanogenerator. Shared data 1: Figure 3(a) data. The output currents of the TENG under different vibration frequencies of external excitation. Shared data 2: Figure 3(b) data. The output voltages of the TENG under different vibration frequencies of external excitation. Shared data 3: Figure 3(c) data. The transferred charge of the TENG when the frequency of external excitation was 2.3 Hz. Shared data 4: Figure 3(d) data. The transferred charge of the TENG under different vibration frequencies of external excitation. Shared data 5: Figure 3(e) data. The peak power-resistance curve of TENG. Shared data 6: Figure 3(f) data. The charging behavior of the TENG. Shared data 7: Figure 4(a) data. The induced currents in the coil of the EMG under different vibration frequencies of external excitation. Shared data 8: Figure 4(b) data. The induced voltages in the coil of the EMG under different vibration frequencies of external excitation. Shared data 9: Figure 4(c) data. The peak power-resistance curve of EMG. Shared data 10: Figure 4(d) data. The charging capability of the EMG. Shared data 11: Figure 5(a) data. The output currents of the TENG in different wave heights. Shared data 12: Figure 5(b) data. The output voltages of [file 5963293.f1.zip › supplementary material-revised (1).pdf]

Supplementary Material

A Non-Resonant Hybridized Electromagnetic-Triboelectric Nanogenerator for Low-Frequency and Irregular Blue Energy Harvesting

Weibo Xie[1,3†], Lingxiao Gao[2,3†], Lingke Wu[4†], Xin Chen[2,3], Fayang Wang[3], Daqiao Tong[3], Jian Zhang[1], Jianyu Lan[7], Xiaobin He[7]*, Xiaojing Mu[1,3]*, Ya Yang[2,5,6]*

[1]State Key Laboratory of Mechanical Transmissions, Chongqing University, Chongqing 400044, China.

[2]CAS Center for Excellence in Nanoscience, Beijing Key Laboratory of Micro-nano Energy and Sensor, Beijing Institute of Nanoenergy and Nanosystems, Chinese Academy of Sciences, Beijing, 100083, China.

[3]Key Laboratory of Optoelectronic Technology & Systems Ministry of Education, International R & D center of Micro-nano Systems and New Materials Technology, Chongqing University, Chongqing 400044, China.

[4]College of Aerospace Engineering, Chongqing University, Chongqing 400044, China.

[5]School of Nanoscience and Technology, University of Chinese Academy of Sciences, Beijing, 100049, P. R. China.

[6]Center on Nanoenergy Research, School of Physical Science and Technology, Guangxi University, Nanning, Guangxi 530004, P.R. China.

[7]Shanghai Academy of Spaceflight Technology, Shanghai Institute of Space Power Source, Shanghai 200245, China.

†These authors contributed equally to this work.

*Corresponding author. Email: hxiaobin1976@163.com(X.H.); mxjacj@cqu.edu.cn(X.M.); yayang@binn.cas.cn(Y.Y.).

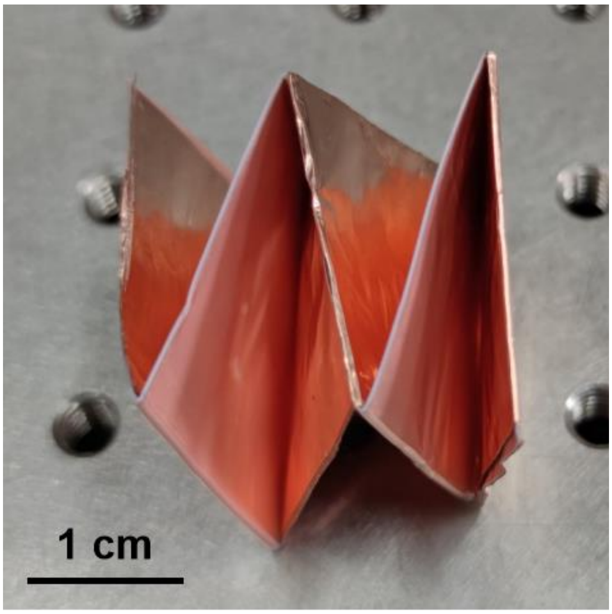

FIGURE S1: Photograph of the TENG.

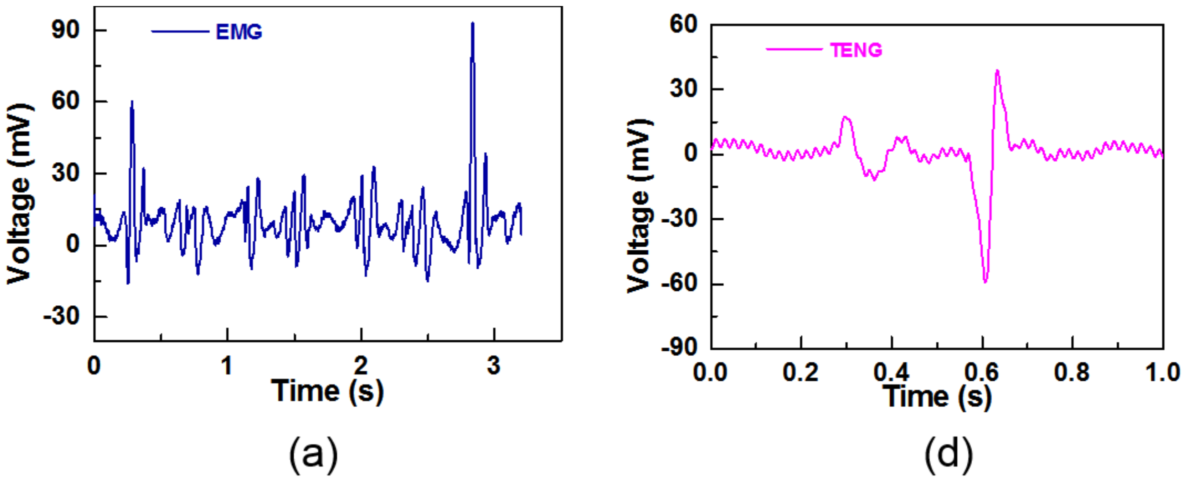

FIGURE S2: The hybridized nanogenerator for wave energy harvesting in Jialing River. (a) The outputs of EMG in Jialing River. (b) The outputs of TENG in Jialing River.

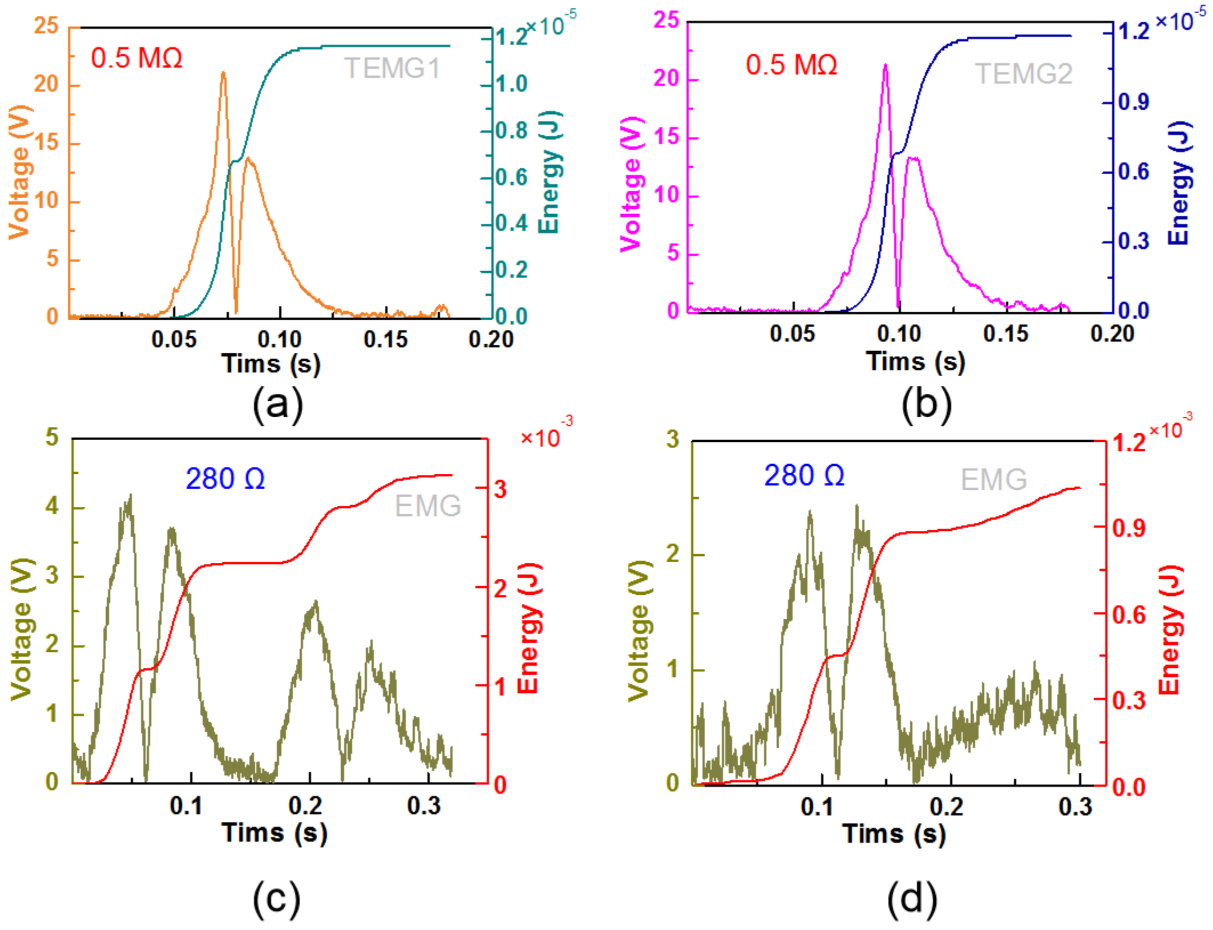

FIGURE S3: The energy harvesting efficiency of the hybridized nanogenerator. (a) The energy captured by TENG1. (b) The energy captured by TENG2. (c) The energy captured by EMG.(d) The energy captured by EMG when the system without TENG.

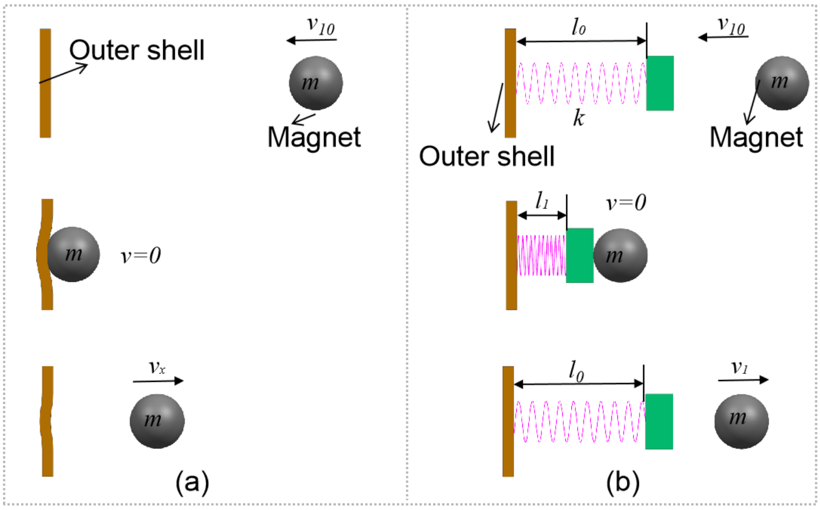

FIGURE S4: The collision model. (a) The collision model of the system without TENG. (b) The collision model of the system with TENG.

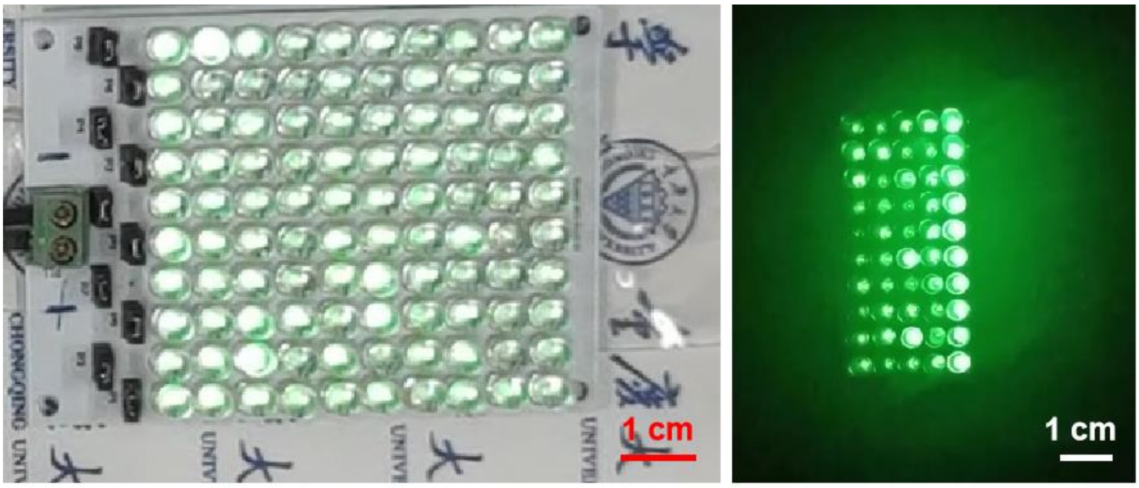

(a) (b)

FIGURE S5: LEDs was lighted up by the hybridized nanogenerator. (a) 100 LEDs in parallel can be lighted up by the EMG. (b) 50 LEDs in series can be lighted up by the TENG.

Research data

Shared data 1: Figure 3(a) Data. The output currents of the TENG under different vibration frequencies of external excitation.

Shared data 2: Figure 3(b) Data. The output voltages of the TENG under different vibration frequencies of external excitation.

Shared data 3: Figure 3(c) Data. The transferred charge of the TENG when the frequency of external excitation was 2.3 Hz.

Shared data 4: Figure 3(d) Data. The transferred charge of the TENG under different vibration frequencies of external excitation.

Shared data 5: Figure 3(e) Data. The peak power-resistance curve of TENG.

Shared data 6: Figure 3(f) Data. The charging behavior of the TENG.

Shared data 7: Figure 4(a) Data. The induced currents in the coil of the EMG under different vibration frequencies of external excitation.

Shared data 8: Figure 4(b) Data. The induced voltages in the coil of the EMG under different vibration frequencies of external excitation.

Shared data 9: Figure 4(c) Data. The peak power-resistance curve of EMG.

Shared data 10: Figure 4(d) Data. The charging capability of the EMG.

Shared data 11: Figure 5(a) Data. The output currents of the TENG in different wave heights.

Shared data 12: Figure 5(b) Data. The output voltages of the TENG in different wave heights.

Shared data 13: Figure 5(c) Data. The induced currents in the coil of the EMG in different wave heights.

Shared data 14: Figure 5(d) Data. The induced voltages in the coil of the EMG in different wave heights.

Shared data 15: Figure 5(f) Data. The induced voltage in the coil of the EMG under a single excitation.

Shared data 16: Figure 6(b) Data. The charging performance of the hybridized nanogenerator for a capacitor of 2000 μF.
